# Intracranial Anatomical Triangles: A Comprehensive Illustrated Review

**DOI:** 10.7759/cureus.1741

**Published:** 2017-10-04

**Authors:** Doniel Drazin, Joy MH Wang, Fernando Alonso, Daxa M Patel, Andre Granger, Mohammadali M Shoja, Marios Loukas, Rod J Oskouian, R. Shane Tubbs

**Affiliations:** 1 Department of Neurosurgery, Cedars Sinai Medical Center; 2 Department of Anatomical Sciences, St. George's University School of Medicine, Grenada, West Indies; 3 Neurosurgery, University Hospitals of Cleveland, Case Medical Center; 4 Neurosurgery, University of Alabama at Birmingham; 5 Department of Anatomical Sciences, St. George's University School of Medicine, Grenada, West Indies; 6 Department of Surgery, University of Texas Medical Branch at Galveston; 7 Neurosurgery, Complex Spine, Swedish Neuroscience Institute; 8 Neurosurgery, Seattle Science Foundation

**Keywords:** anatomy, skull base, neurosurgery, landmarks, skull base triangles

## Abstract

There are multiple anatomical triangles of the skull base. However, to our knowledge, there has been no comprehensive review of these geometric landmarks. To allow for a safe and consistent approach to lesions of the skull base such as those near the internal carotid artery, internal acoustic meatus, and cavernous sinus, a comprehensive review of the variations with illustrations is required. This article provides an overview of the anatomical borders, dimensions, and surgical implications as well as illustrations of the major skull base triangles.

## Introduction and background

Owing to its location and formidable anatomical structures, the skull base is a technically difficult region for neurosurgeons, and many surgical approaches to this area have yet to be perfected. Parkinson first described a triangle in the lateral wall of the cavernous sinus in the mid-1960s' [1-2]. Dolenc further elaborated on direct microsurgical approaches in terms of the anatomy and surgery within the corridors of the cavernous sinus [3-7]. Since then, the oculomotor, Glassock’s and Kawase’s triangles have been documented, but details of the other triangles are yet to be reported [8-9]. In addition, the names and descriptions of the triangular spaces are inconsistent [10-12].

Understanding the normal anatomy of the four cavernous triangles, four middle fossa triangles, and two paraclival triangles are critical for approaching surgical lesions around the cavernous sinus, petrous and cavernous part of the internal carotid artery, internal acoustic meatus, and the labyrinth. This manuscript will review the anatomical borders, average dimensions, and surgical implications of these triangles and will provide illustrations to aid in understanding their locations.

## Review

Triangles of the cavernous sinus

**Figure 1:**
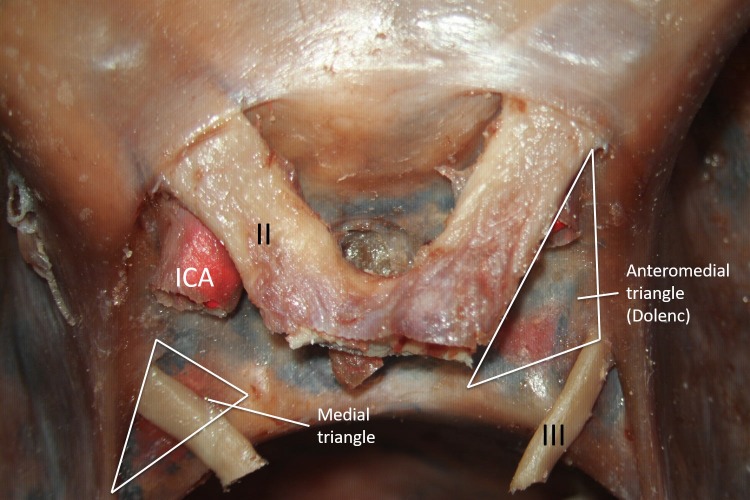
Cadaveric dissection of the sellar region. The left supraclinoid segment of the internal carotid artery (ICA) is seen. Acess to the cavernous ICA can be obtained via the anteromedial triangle. The medial triangle’s primary structure is the oculomotor nerve (III) Legend: II - optic nerve; III - oculomotor nerve; ICA - internal carotid artery (original image).

The image shows the anteromedial triangle (Figure 1), also known as the clinoidal triangle or Dolenc’s triangle. Its borders are the optic nerve, oculomotor nerve, tentorial edge (and the dura extending between the dural entry point of the third cranial nerve and the optic nerve) [12]. Its contents are the clinoidal internal carotid artery (ICA) and the anterior clinoid process.

Drilling the anterior clinoid process, either extradurally or intradurally exposes the clinoidal or anteromedial triangle area. It is bordered by the optic nerve medially, oculomotor nerve laterally, and the tentorial edge extending between the second and third cranial nerves at the base. Watanabe, et al. found that the average measurements of the medial border, lateral border, base, and the area are 9.5, 13.3, 7.2 mm and 32.3 mm^2^, respectively [12]. These dimensions have been further explored via microsurgical techniques on cadaveric dissections and the average sizes of the medial border, lateral border, base, and area are 7.34, 13.89, 8.53 mm and 26.25 mm^2^, respectively [13]. Day and Fukushima described the anterior triangle as defined by the lateral border of the optic nerve, the medial wall of the superior orbital fissure dura, and the dural ring surrounding the internal carotid artery (ICA) as it enters the subarachnoid space. According to these borders, they measured the dimensions as 6.30 posteriorly, 6.88 medially, and 8.72 mm laterally [10-11]. Although these various authors differ in their measurements, they agree about this triangle’s content: the clinoidal segment of the ICA in the middle, optic strut anteriorly, and the roof of cavernous sinus posteriorly. To reach the clinoidal ICA, the anterior clinoid process can be pneumatized so that the clinoidal ICA can be visualized [14]. The anterior clinoid process can also be drilled to obtain access to the sphenoidal sinus. The subclinoidal ICA is occasionally surrounded by an osseous bridge between the anterior and middle clinoid process, and removing this process in one piece has been reported to injure the ICA [15]. Furthermore, with a meningioma invading the anterior clinoid process, it becomes necessary to perform an intradural resection via the clinoidal triangle [16].

The clinoidal triangle can be exposed via the endoscopic supraorbital extradural approach. Following a supraorbital craniotomy and the drilling of the sphenoidal ridge, the orbital roof and the greater and lesser sphenoid wings, the floor of the middle cranial fossa can be exposed by lifting the temporal dura slightly. At this point, the anterior clinoid process can be removed to allow the clinoidal triangle to be visualized [17]. Through the endoscopic endonasal approach, Komatsu proposed that the clinoidal triangle along with the anteromedial and anterolateral triangles is a possible route to the middle cranial fossa [18].

The medial triangle also is known as the oculomotor triangle or the Hakuba’s triangle is shown in Figure 1. Its borders are the anterior petroclinoid dural fold, posterior petroclinoid dural fold, and the interclinoid dural fold. Its contents are the oculomotor Nerve and the ICA (horizontal segment).

In exploring the oculomotor nerve and the horizontal segment of the ICA, the oculomotor, medial or Hakuba’s triangle is approached [19]. It is one of the few triangles formed by the superior wall of the cavernous sinus and dural folds: medially by the interclinoid fold, laterally by the anterior petroclinoid fold and the base by the posterior petroclinoid fold. The average measurements of the medial, lateral, and base sides are 10.4, 16.1, 12.2 mm, respectively. The oculomotor triangle is significant for accessing tumors located in the medial cavernous sinus and for interpeduncular lesions. Day, et al. described this triangle with different corners: subclinoidal carotid segment, posterior clinoid process, and the porus oculomotorius, calling it the medial triangle [10]. The dimensions of the medial triangle differ by a few millimeters: 9.6 x 16.6 x 13.8 mm [14, 20]. Through this triangle’s window, the horizontal portion of the cavernous ICA can be explored for various vascular pathologies. It also has a very important relationship with aneurysms that form at the junction of the internal carotid artery and the posterior communicating artery [21]. Also visualized are the posterosuperior venous space of the cavernous sinus, the posterior bend of the intracavernous ICA with the origin of the meningohypophyseal trunk, the initial intracavernous course of the abducens nerve, and the interclinoid ligament [14].

The third and fourth cranial nerves enter the cavernous sinus in this area of the superior wall. The average distance from the dural foramen of the occipital nerve to the anterior clinoid process was 7.2 mm and to the posterior clinoid process was 8.5mm. The dural opening for the trochlear nerve was on average, 16.04 mm and 12.74 mm away from the anterior and posterior clinoid processes, respectively [14].

**Figure 2:**
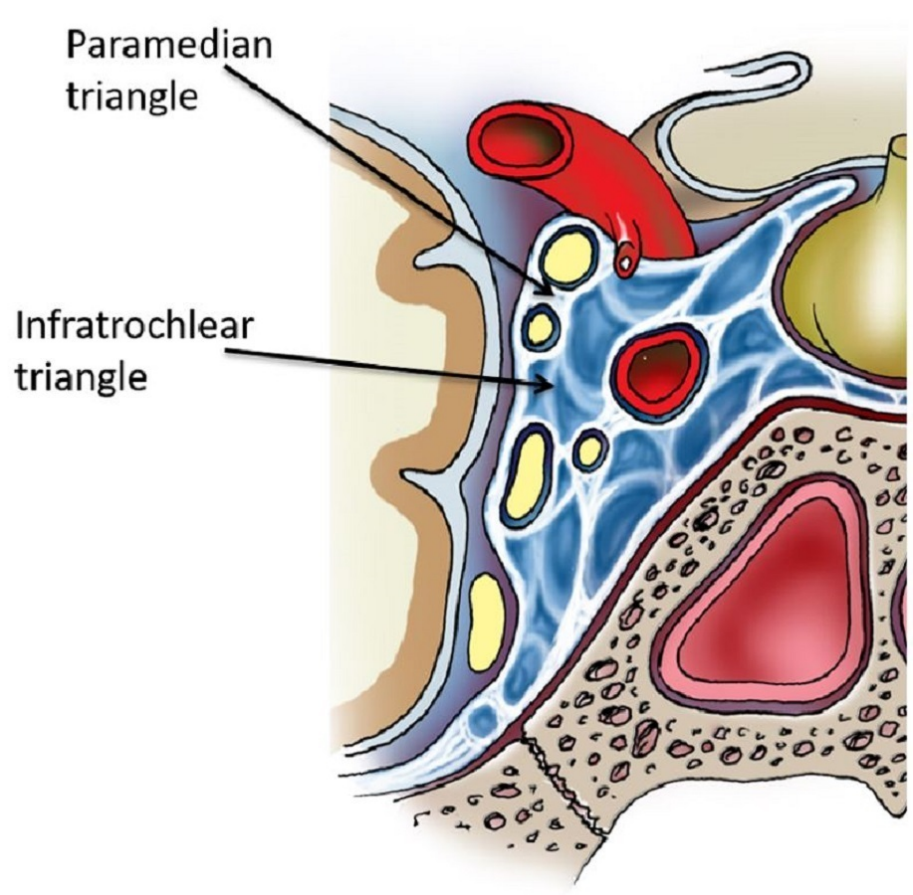
Coronal section through the cavernous sinus (shown in blue). The paramedian triangle allows access between the oculomotor and trochlear nerves and the infratrochlear triangle allows access between the trochlear and V1 nerves. The abducens nerve is seen medal to V1 and the cavernous internal carotid artery is seen in cross-section (original illustration).

The image shows paramedian triangle (Figure 2), also known as the supratrochlear triangle. Its borders are the oculomotor nerve, trochlear nerve, tentorial edge (and the dura extending between the dural entry points of the third and the fourth cranial nerves) [12]. Its contents are the meningohypophyseal trunk.

Following the posterior bend of the ICA, it allows the supratrochlear or paramedian triangle to be entered. This triangle’s medial border is the oculomotor nerve, the lateral border is the trochlear nerve and the base is formed by the tentorial edge extending between the third and fourth cranial nerves. In addition to the meningohypophyseal trunk origin, the inferolateral trunk and less commonly the medial loop of the ICA are identified in this space [3, 10]. The dimensions of the medial, lateral, and base are 13.18, 14.27, and 5.51 mm, respectively. These measurements correlate with Watanabe’s documentation of 10.9, 14.0, and 7.0 mm, respectively [12]. This triangle is used to explore the medial loop of the intracavernous ICA and the meningohypophyseal trunk and is accessed during clipping of cavernous aneurysms.

**Figure 3:**
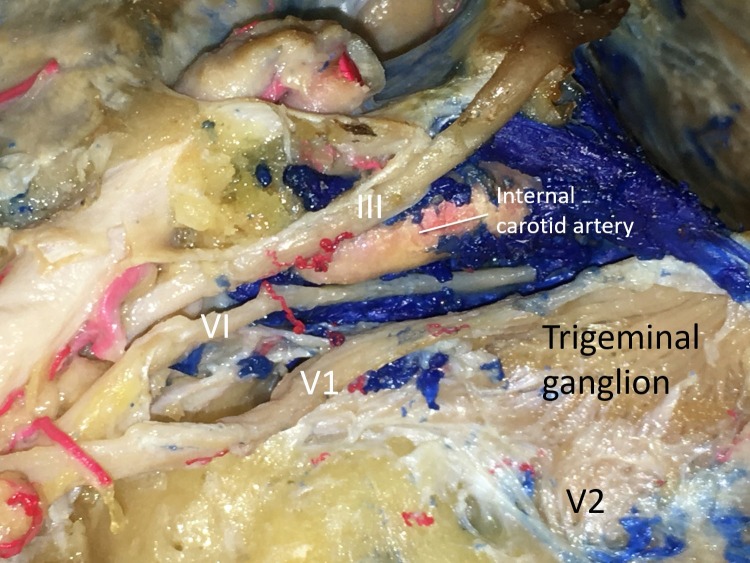
Left lateral dissection of the cavernous sinus Figure illustrating important neurovascular structures encountered during surgical approaches using the various cavernous sinus triangles Legend: III - oculomotor nerve; VI - abducens nerve; V1 - the ophthalmic branch of the trigeminal nerve; V2 - the maxillary branch of the trigeminal nerve (original image).

The image shows the infratrochlear triangle (Figure 2), also known as the Parkinson’s triangle, superolateral space by Watanabe, et al. [12]. Its borders are the trochlear nerve, ophthalmic division (V1) and the tentorial edge. Its contents are the ICA (cavernous) and the abducens nerve (Figure 3).

To enter the cavernous sinus directly, Parkinson originally described the infratrochlear or Parkinson’s triangle [1, 22]. Watanabe and Day refer to it as the superolateral space [10, 12]. It is limited medially by the trochlear nerve, laterally by the ophthalmic division of the trigeminal nerve and at the base of the tentorial edge of these two nerves. Although few other studies mention variations of the measurements, Watanabe documented the dimensions as 13.1, 16.4, and 6.2 mm for the medial, lateral, and base of Parkinson’s space respectively [10, 12, 23-24]. This triangular area is relatively narrow, but it can be enlarged during surgical approaches via retraction or dissection of the trochlear nerve medially and the trigeminal nerve laterally [6]. Further opening of this space allows the entire intracavernous segment of the ICA to be explored from lateral ring to proximal ring. In addition, it permits the sixth nerve to be completely exposed from its entry through Dorello’s canal to its exit through the superior orbital fissure [6]. Thus, this triangle is valuable in the surgical management of vascular abnormalities along the ICA as well as cranial nerve mass lesions. Knosp, et al. discussed how Parkinson’s triangle can be used to access and resect meningiomas occupying the cavernous sinus [25].

After exposure of the lateral wall of the cavernous sinus via the endoscopic supraorbital extradural approach, Parkinson’s triangle can be visualized with slight temporal retraction. The number of structures that can be visualized via this approach reflects its great utility in microsurgery. The posteroinferior part of the cavernous sinus can be exposed through this triangle along with lateral views of the posterior vertical segment, posterior bend, horizontal segment of the ICA, branches of the ICA, and the posterior wall of the cavernous sinus [17].

The parasellar region in infants has rarely been discussed in the literature. Virtually all of the information available about this region has come from studies of adult specimens. Weninger, et al. used 49 specimens from human infants aged zero to nine months in their study of the parasellar region. The measurement along the upper rim of the trigeminal nerve side of Parkinson’s triangle was 7.33 mm, and along the clivus side was 3.77 mm. The average height at the level of the posterior clinoid process was 3.48 mm. In 50% of the cases examined, the meningohypophyseal trunk, its branches, the site of its origin from the internal carotid artery, and the medial wall of the upper posterior portion of the parasellar region were approachable through this triangle in infants [26].

Triangles of the middle fossa

**Figure 4:**
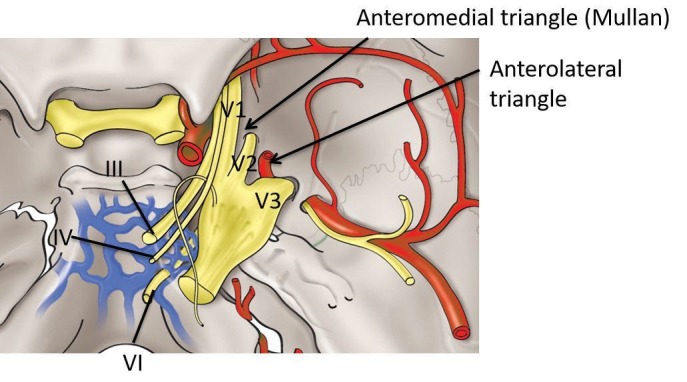
Schematic drawing of the middle fossa approaches using the anteromedial and anterolateral triangles Legend III - oculomotor nerve; IV - trochlear nerve; VI - abducens nerve; V1 - the ophthalmic branch of the trigeminal nerve; V2 - the maxillary branch of the trigeminal Nerve; V3 - the mandibular branch of the trigeminal nerve (original illustration).

The image shows the anteromedial triangle (Figure 4), also known as the anteromedial middle fossa triangle and the Mullan’s triangle. Its borders are the ophthalmic nerve (V1), maxillary nerve (V2) and the superior orbital fissure to foramen rotundum. Its contents are sphenoid sinus, superior ophthalmic vein and the abducens nerve.

Also referred to as Mullan’s triangle, the anteromedial triangle’s boundaries are formed by the ophthalmic division of the trigeminal nerve medially and the maxillary division of the trigeminal nerve laterally [27]. The base of the triangle consists of the anterolateral wall of the bony middle cranial fossa formed by a line connecting the superior orbital fissure to the foramen rotundum. The lengths of these limits are 11.7 x 7.8 x 12.0 mm [12]. This corridor is well suited for exposing several important structures, including the superior orbital vein, sixth cranial nerve, sphenoid sinus, and the ophthalmic vein. Further dissection within Mullan’s space allows for access to carotid-cavernous fistulas. For exposure of this triangle during endoscopic surgery via the supraorbital extradural approach, slight temporal retraction is necessary [17].

Cranial nerve deficits following intracavernous surgical interventions can occur as a result of direct manipulation of the nerves or disturbance of their blood supply. The superior orbital fissure artery, usually branching from the inferolateral trunk, runs in the lateral wall of the cavernous sinus and supplies cranial nerves III, IV, VI, and ophthalmic (V1), the ophthalmic division of the trigeminal nerve. Hence, procedures that interrupt the anatomy of the lateral wall of the cavernous sinus at the anteromedial triangle places the vascular supply of these nerves at risk [28], though collaterals do exist [29].

The image shows the anterolateral triangle (Figure 4), also known as the anterolateral middle fossa triangle and the lateral triangle. Its borders are the maxillary nerve (V2), mandibular nerve (V3), and the foramen rotundum to foramen ovale. Its contents are the lateral sphenoid wing, sphenoid emissary vein, and the cavernous-pterygoid venous anastomosis.

The anterolateral triangle is formed medially by the maxillary division and laterally by the mandibular division of the trigeminal nerve [13]. The base is identified via a bony line connecting the foramen rotundum and foramen ovale. The border dimensions are 13.9, 15.5, and 7.6 mm representing the medial, lateral, and base of the triangle respectively [12]. This space is used to expose the lateral sphenoid wing, sphenoidal emissary vein, and cavernous-pterygoid venous anastomosis. Through this window, lesions located in the lateral part of the cavernous sinus are uncovered.

Kadera, et al. demonstrated that sufficient access to resect tissue from intracavernous lesions could be gained via the anterolateral triangle through a frontotemporal intradural approach without a zygomatic or orbitozygomatic osteotomy. This approach required only minimal dissection of the dura propria and inner layer, avoiding excessive retraction of the temporal lobe [30].

**Figure 5:**
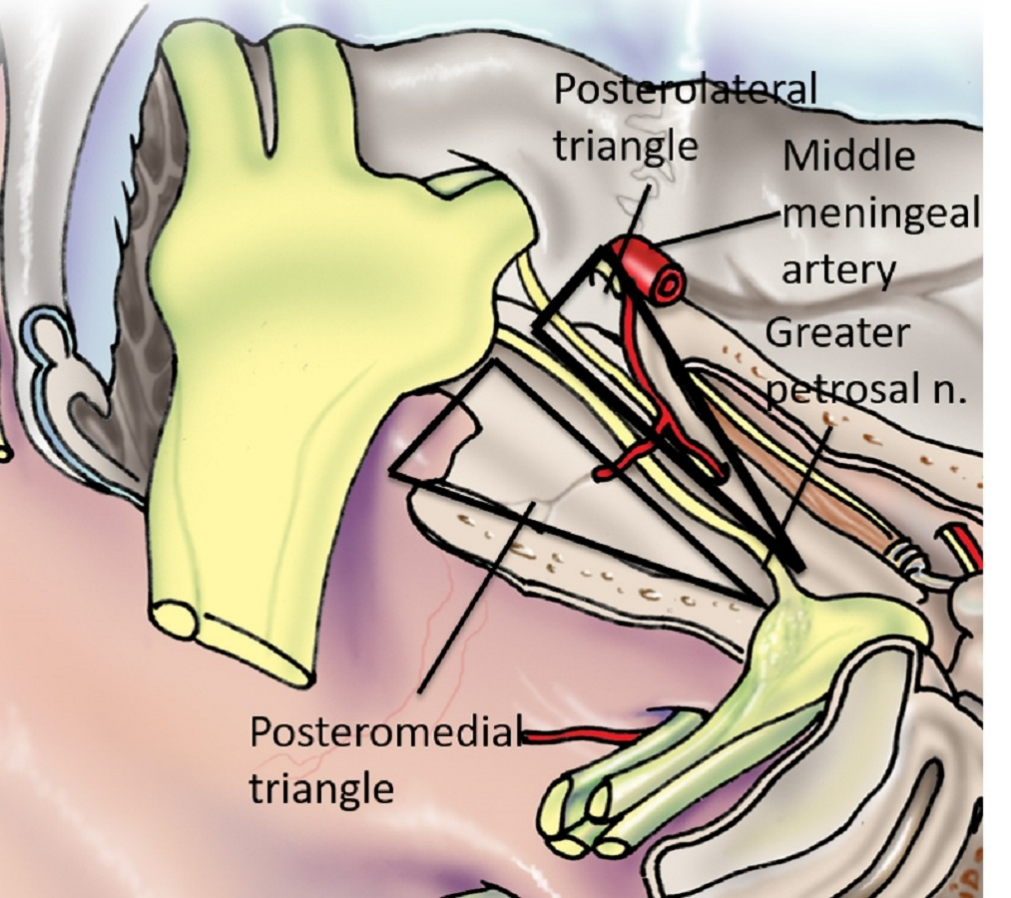
Schematic drawing of the salient anatomy of the posteromedial and posterolateral triangles (original illustration).

**Figure 6:**
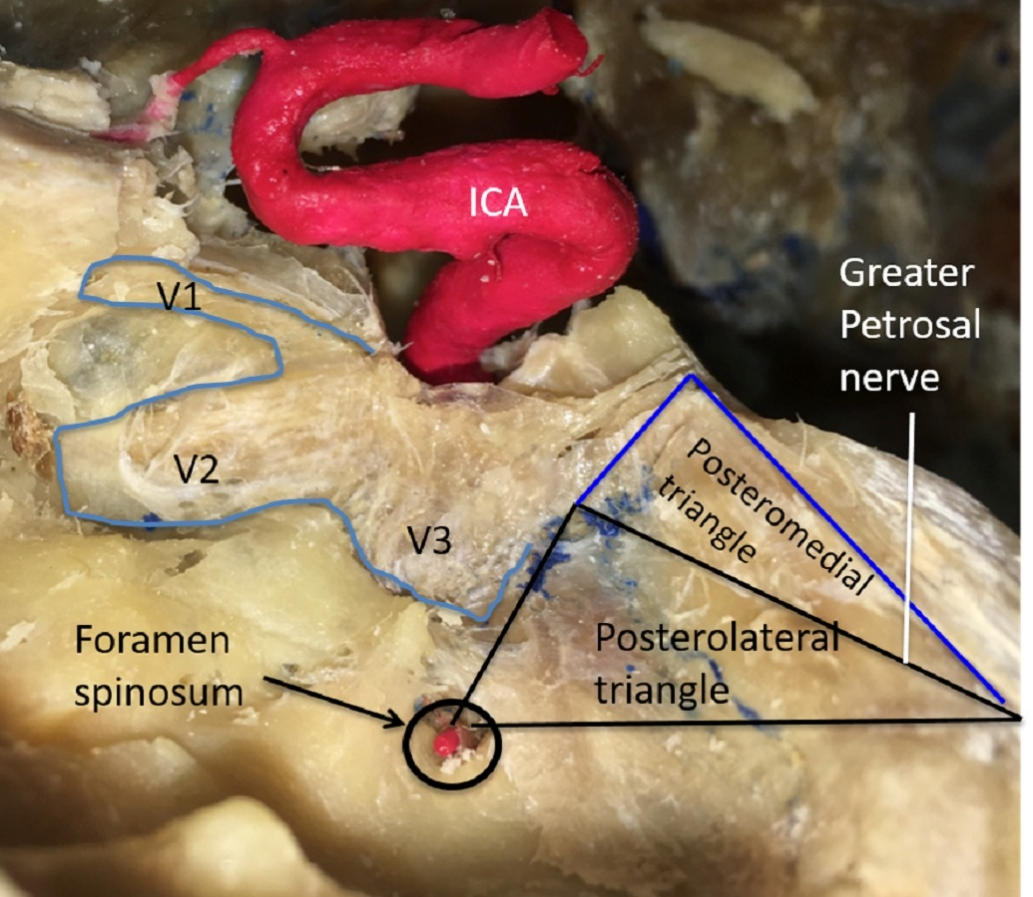
Left lateral dissection of the salient anatomy of the posteromedial and posterolateral triangles. Legend: ICA - internal carotid artery; V1 - the ophthalmic branch of trigeminal Nerve; V2 - the maxillary branch of trigeminal nerve; V3 - the mandibular nerve of the trigeminal nerve (original image).

The image shows the posteromedial triangle (Figures 5-6), also known as the Kawase’s triangle, Kawase-Shiobara’s triangle, and the rhomboid space. Its borders are the greater petrosal nerve, mandibular nerve (V3), arcuate eminence and the superior petrosal sinus. Its contents are the petrous apex, ICA, and the vertebrobasilar junction.

Kawase first described this rhomboid-shaped space, possibly the largest of all of the triangles referred to as the posteromedial or Kawase triangle [9]. Its boundaries are the lateral margin of the greater petrosal nerve medially and the lateral edge of the trigeminal nerve at the crest of the petrous apex laterally. The base consists of a line connecting the posterior border of the mandibular division of the trigeminal nerve and the crest of the petrous apex to the center of the geniculate ganglion. Watanabe documented this triangle’s dimensions as 16.1 x 13.9 x 12.5 mm and further commented on the consistency of the shape of this space [12]. Removing the bone in this triangle permits the petrous apex or petroclival area and internal acoustic meatus to be exposed. This section of petrous bone does not contain vascular or neural structures and can be drilled safely. The corridor through Kawase’s space allows access to the vertebrobasilar junction, the root of the trigeminal nerve, and anterolateral brain stem.

In 2015, Tripathi, et al. conducted a volumetric analysis of 12 dried temporal bones using laser technology [31]. They defined the bounds of Kawase’s triangle as follows: (1) the junction of the greater petrosal nerve with the lateral border of the mandibular division of the trigeminal nerve (V3), (2) the lateral margin of the porus trigeminus, and (3) the anteromedial margin of the arcuate eminence. They compared the dimensions of this triangle to that of a modified Dolenc-Kawase (MDK) rhomboid with the following boundaries: (1) the lateral margin of V3 after medial mobilization of the trigeminal nerve, (2) the bony surface just lateral to the petroclival ligament, and (3) the arcuate eminence and the intersection of a line drawn along the axis of the greater petrosal nerve and the arcuate eminence. Tripathi, et al. determined that the area of Kawase’s triangle averaged 269.13 mm^2^ with a mean volume of 666.61 mm^3^ [31].

The image shows the posteromedial triangle (Figures 5-6), also known as the Glasscock’s triangle. Its borders are the mandibular nerve (V3), greater petrosal nerve and the foramen spinosum to arcuate eminence. Its contents are the foramen spinosum, horizontal petrous ICA and the infratemporal fossa.

The posterolateral triangle is relatively inconsistent in size and is bounded laterally by a line from the foramen spinosum to the arcuate eminence of the petrous bone and medially by a line between where the greater petrosal nerve crosses under the mandibular division of the trigeminal nerve and the foramen spinosum [8]. Its base is the greater petrosal nerve itself [13]. Glasscock originally defined the corners as the posterior rim of the foramen ovale, the apex of the cochlea, and the posterior border of the mandibular division of the trigeminal nerve [8]. Rhoton defined this triangle by the lateral surface of the trigeminal nerve and by the anterior margin of the petrosal nerve [32]. Contrary to Watanabe’s and Dolenc’s measurements, Isolan documented the dimensions as 14.7x 7.04 x 15.3 mm, with 49.2 mm^2^ area [3, 12]. Isolan’s parameters can be reproduced more precisely than the arcuate eminence. Day’s measurements showed the medial border to be bigger than Isolan’s and Watanabe’s, with the lateral border as smaller, owing to differences in the anatomical points among the authors [10]. Glasscock’s triangle is known not only for variations in its size, but also its contents, which include the greater and lesser petrosal nerves, tensor tympani muscle, Eustachian tube, middle meningeal artery, foramen spinosum, infratemporal fossa, and most importantly, the horizontal petrous ICA [6]. Drilling away the Glasscock’s triangle exposes the horizontal intra-petrosal segment of the ICA for proximal control for a bypass graft or anastomosis, and also allows access to the cavernous sinus for the tumor resection [33].

Triangles of the paraclival region

**Figure 7:**
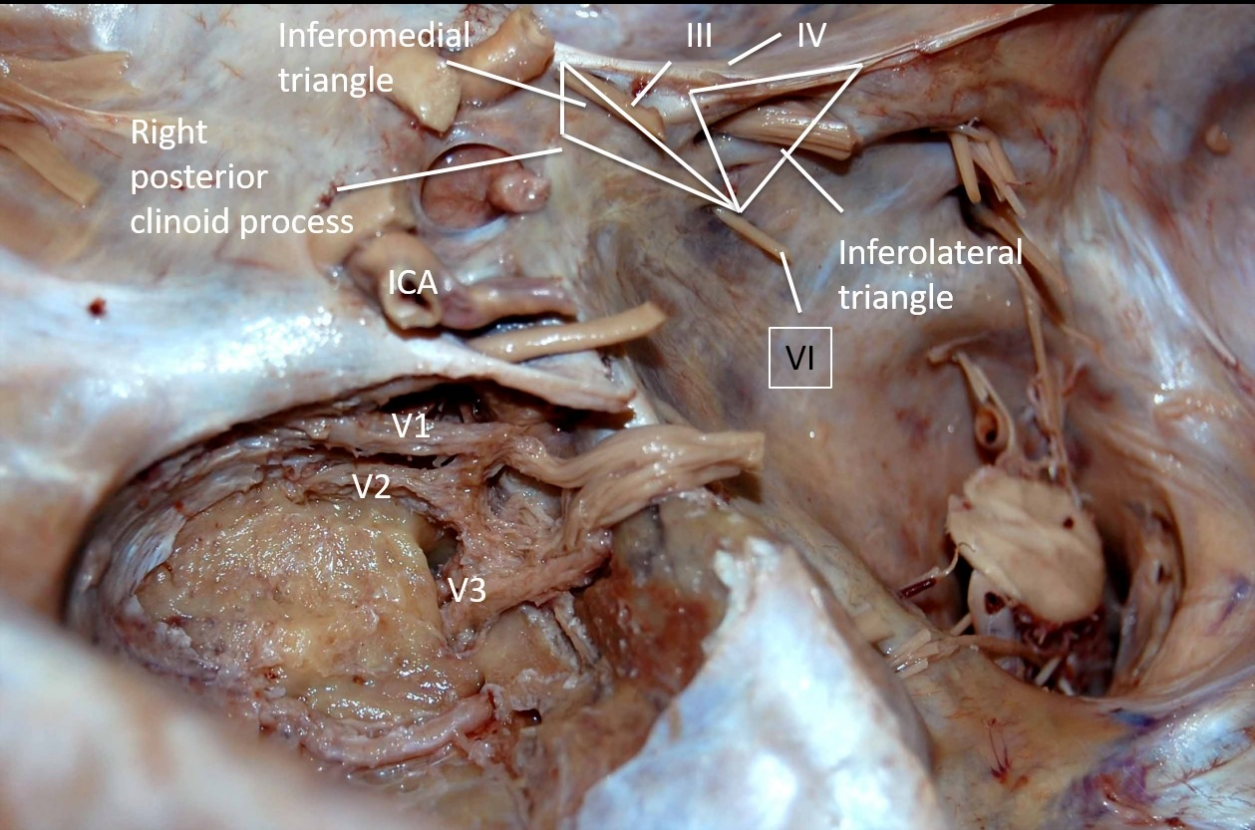
Left posterolateral dissection of the anatomy of the inferomedial and inferolateral triangles. Legend: ICA - internal carotid artery; V1 - the ophthalmic branch of trigeminal nerve; V2 - the maxillary branch of trigeminal nerve; V3 - the mandibular nerve of the trigeminal nerve; III - oculomotor nerve; IV - trochlear nerve; VI - abducens nerve (original image).

The image shows the inferomedial triangle (Figure 7), also known as the inferomedial paraclival triangle. Its borders are shown as the line from the dural entries of the trochlear and abducens nerves, a line from the dural entries of the abducens nerve and posterior clinoid and the petrous apex. Its contents are the porous abducens (Dorello’s canal) and Gruber’s ligament.

The inferomedial paraclival triangle is defined medially by a line connecting the dural entrance of the sixth cranial nerve at Dorello’s canal to the posterior clinoid process, laterally by a line connecting the dural entrance of the fourth and sixth cranial nerves and at the base by the petrous apex. The measurements according to Isolan are 16.2 x 11.4 x 7.3 mm, with 34.9 mm^2^ area, which corresponds to measurements from other cadaveric studies [12]. The inferomedial space extends into the posterior cavernous sinus wall and drilling the medial part of this space exposes the lateral edge of the dorsum sellae, petroclival fissure, and Dorello’s canal. Furthermore, this triangle contains the abducens nerve, posterior genu of the ICA, the dorsal meningeal branch of the meningohypophysial trunk, basilar venous plexus and overlying dura, posterior petroclinoid fold and Gruber’s petrosphenoid ligament.

The image shows the inferolateral triangle (Figure 7), also known as the inferolateral paraclival triangle. Its borders are the line from the dural entries of trochlear and abducens nerves, a line from the dural entries of the abducens nerve and the petrosal vein and the petrous apex. Its contents are the porous trigeminii (the Meckel’s cave).

The inferolateral paraclival triangle is defined medially by a line between the dural entrance of the trochlear nerve into the tentorium cerebelli to the dural entry of the abducens nerve and laterally by a line between the dural entry point of the abducens nerve and the petrosal vein. This triangle shares the same base as the inferomedial triangle, the petrous apex. Isolan measured 11.4 mm medially x 15.0 mm laterally x 7.9 mm base, with a 34.9 mm^2^ area [12]. Dolenc divided this space by a line formed between the entry point of the petrosal vein and the entry point of the fifth cranial nerve; relative to the fifth cranial nerve, the tentorial triangle is above and the osseous triangle is below [3-5]. Access to the tentorial artery, superior petrosal sinus, and the Meckel’s cave is obtained through the tentorial triangle, while the fifth cranial nerve is encountered from the osseous triangle.

Triangles of the sellar region

The image shows the preinfundibular triangle (Figure 7). Its borders are the right and left optic nerves and its contents are the sella.

Prescher, et al. used reproducible and reliable landmarks in the Sella region to define three triangles on 48 adult skull base specimens [34].

The preinfundibular triangle is located anterior to the sellar region. Two lines, both drawn from the point where the pituitary stalk penetrates the dura to the medial entrances of the right and left optic nerves, form the right and left borders, respectively (Note that all orientations are from a posterior view). The anterior border is formed by the line connecting the medial points of each optic nerve before entering the optic canal. The average lengths of these borders in mm are 12.3, 12.6 and 13.8 for the right, left and anterior borders, respectively [34].

**Figure 8:**
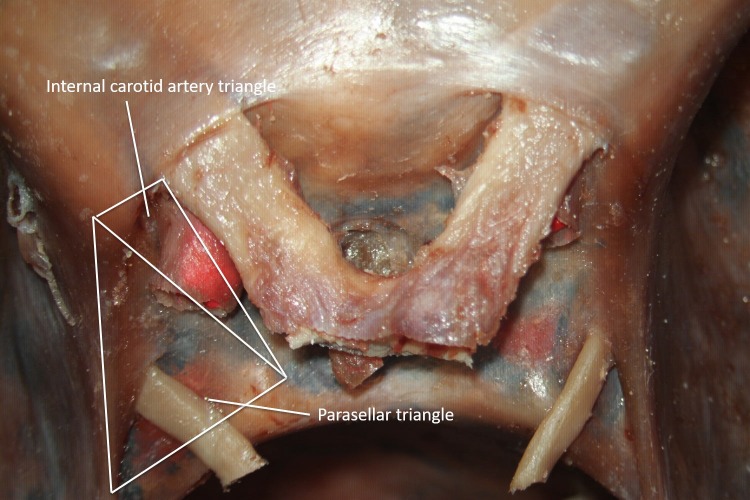
Superior view of the parasellar and internal carotid artery triangles (original image).

The image shows the parasellar triangle (Figure 8). Its borders are the anterior and the posterior petroclinoid dural fold, tentorial edge and the anterior and posterior clinoid process. Its contents are the sella turcica.

The parasellar triangle can be found in the area just lateral to the sella. The medial border is formed by the length of the anterior petroclinoid dural folds, from the tentorial edge to the anterior clinoid process. The length of the posterior petroclinoid fold, from the ventromedial tentorial edge to the posterior clinoid process, makes up the lateral border. The ventral border is formed by a line connecting the anterior clinoid process to the posterior clinoid process. On average, the length of the medial, lateral and ventral borders on the left of the sella were 12.1, 12.5, and 8.3 mm, respectively. On the right side, the corresponding measurements were 12.4, 12.5, and 8.3 mm in the same order [34].

The image shows the internal carotid artery triangle (Figure 8). Its borders are the anterior and the posterior clinoid process and the optic nerve. Its contents are the sella.

This triangle is found just anterior to the parasellar triangle. Its lateral border is formed by the line connecting the anterior clinoid process to the lateral point of the optic nerve just outside the optic canal. The medial limit is formed by the line connecting the posterior clinoid and the lateral point of the optic nerve just outside the optic canal. The connection between the anterior clinoid process and the posterior clinoid process forms the posterior border. The medial border of this triangle measured an average of 12.2 mm on the left and 12.3 mm on the right. The lateral border measured, on average, 8.0 mm on the left and 8.1 mm on the right. The average measurements of the dorsal border were 8.3 mm on the right and 8.4 mm on the left [34].

Other skull base triangles

The image shows the parapetrosal triangle. Its borders are the spinopterygoidal (Henle’s spine to foramen ovale), bispinal (between the two spines of Henle), midsagittal line (posterior margin of the medial pterygoid plate). Its contents are the spines of Henle.

Ray, et al. investigated the para petrosal triangle and measured the distance from Henle’s spine to various important anatomical structures [22]. The corners of this triangle are formed by the points of intersection of three lines: the spinopterygoidal, bispinal and midsagittal lines. The spinopterygoidal line extends from Henle’s spine of the posterior margin of the medial pterygoid plate through the medial margin of the foramen ovale. The bispinal line runs between the two spines of Henle. The midsagittal line originates from the posterior margin of the medial pterygoid plate and is essentially the perpendicular bisector of the bispinal line.

The average length of the spinopterygoidal line was 54.1 mm on the right and 53.7 mm on the left in both males and females. The average distance from Henle’s spine to the point where the bispinal line crosses the midline was 62.0 mm on the right and 61.2 mm on the left in males. In females, the average distances were 62.3 mm and 61.5 mm on the right and left, respectively [35-36].

The image shows the superior petrosal triangle. Its borders are the foramen spinosum, the root of zygoma and the head of the malleus. Its contents are the bony tegmen over the head of the malleus.

In 1961, House described a middle cranial fossa approach that allowed the entire length of the internal acoustic meatus to be visualized [37-38]. The results of a middle cranial fossa approach carried out on the temporal bones of twenty adults and six consecutive patients were presented by Catalino and Eden. In this paper, they discussed a method for locating the head of the malleus [39].

Miller and Pensak performed an anatomical study using ten preserved human cadaveric temporal bones to define the anatomical relationship between the root of the zygoma (ROZ), the posterolateral lip of the foramen spinosum, and the bony tegmen over the head of the malleus. In this study, they defined a superior petrosal triangle that serves the purpose of reliably locating the bony tegmen over the head of the malleus. The foramen spinosum, the ROZ, and the head of the malleus formed the corners of this triangle. The distance from the ROZ to the head of the malleus was 18.7 mm on average. The average distance from the ROZ to the foramen spinosum was 30.0 mm. These were the two sides of the triangle that had documented measurements. The distance from the foramen spinosum to the head of the malleus measured 19.2 mm. Miller and Pensak, therefore, concluded that the intersection of the two 19 mm arcs drawn from the ROZ and the foramen spinosum approximates the location of the head of the malleus [38].

**Figure 9:**
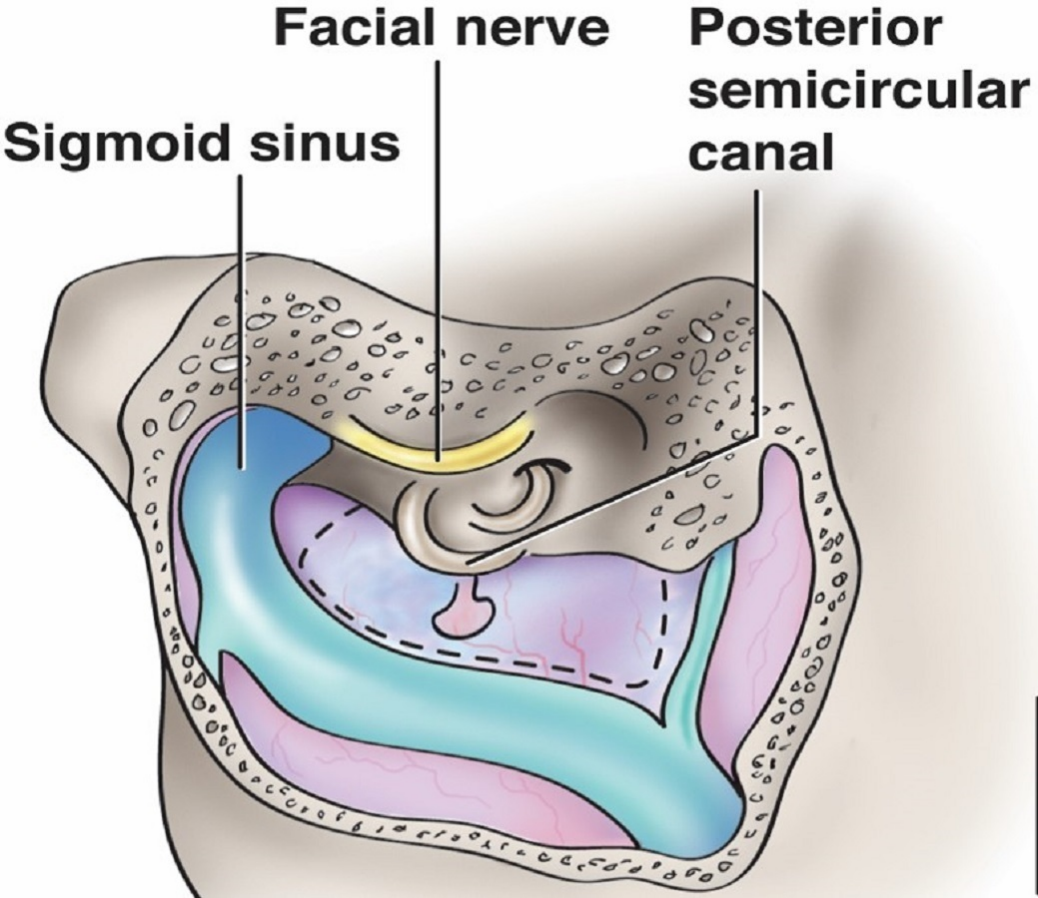
Schematic drawing illustrating the important anatomy of Trautmann’s triangle (dotted region). The tributary draining to the transverse-sigmoid junction to the right is the superior petrosal sinus, which makes up the third leg of the triangle (original illustration).

The image shows the Trautmann’s Triangle (Figure 9). Its borders are the superior jugular bulb, sino-dural angle and the posterior semicircular canal. Its contents are the posterior petrous bone, ventral brainstem and the cranial nerves (CN) CN V, VII and VIII.

Intracranial access via the retrolabyrinthine approach exposes a portion of the dura mater on the posterior side of the temporal bone that faces the cerebellopontine angle. This triangular-shaped area is termed Trautmann’s triangle and is also known as the retromeatal trigone [40]. Much of this area is resected during posterior petrosectomy [41]. This triangle is bordered by the superior jugular bulb at the inferior vertex, the sino-dural angle along the superiorly directed base, and anteriorly by the posterior semicircular canal [40]. Tubbs, et al. classified Trautmann’s triangle into three types on the basis of the area: type one: with an area of less than 75 mm^2^ (37.5%), type two: 75-149 mm^2^ (35%) and type three: with an area of 150 mm^2^ (27.5%) or greater [40]. The size of the operative aperture gained through exposure of Trautmann’s triangle can be affected by the relationship of the jugular bulb to the internal auditory canal. A study of 200 temporal bones revealed an incidence of high jugular bulbs of 16.5%, defined as a jugular bulb at the level of the internal auditory canal or higher [42]. Its clinical applications include the pathophysiology and the surgical management of intractable Ménière’s disease and resection of petroclival meningiomas and other related lesions ventral to the brain stem [40-41, 43].

Magnetic resonance imaging (MRI) and the cavernous sinus triangles

The magnetic resonance imaging (MRI) is typically the preferred method for imaging the central nervous system. Hermann and Sloniewski incorporated MRI technology with cavernous sinus microanatomy [44]. In their manuscript, they defined four areas in the coronal plane and two in the sagittal plane. They then proceeded to list the anatomical triangles that fall into those areas. The defined areas were perpendicular to the plane of the anatomical triangles permitting a “face on” view of the triangles and their contents.

In the first of the areas in the coronal plane, Area coronal plane (C1), the scan line spans from the oculomotor nerve after its entrance to the cavernous sinus to the second cranial nerve. The anteromedial triangle is contained within this area. The main landmark in this area is the anterior clinoid process.

Area C II is again in the coronal scan line from the oculomotor nerve running inside the cavernous sinus to the upper edge of the Meckel’s cave. The paramedial and Parkinson’s triangles are located in this area.

Area C III consists of the lower section of the lateral wall of the cavernous sinus. This corona area spans from the lateral wall of the Meckel’s cave and the lower part of the lateral wall of the cavernous sinus. The lateral and anterolateral triangles are contained in this region.

Area C IV in the coronal scan consists of the superior edge of the petrosal bone starting from its apex to the internal carotid artery in the foramen lacerum. The main landmark in this area is the internal carotid artery in the carotid canal running forward to the foramen lacerum. The petrosal bone can also be seen here. This area contains the posteromedial and posterolateral triangles.

Two areas were defined in the sagittal plane. The first, Area sagittal plane (S I), comprises the superior wall of the cavernous sinus from the anterior clinoid process to the posterior clinoid process and the petrosal bone apex. The oculomotor nerve entering the superior wall of the sinus is the main landmark in this area. The oculomotor triangle is found here.

In the second area in the sagittal plane, Area S II, are the posterior wall of the cavernous sinus and the posterior edge of the petrosal bone starting from the posterior clinoid process to the internal acoustic canal. This area contains the inferomedial and inferolateral triangles.

## Conclusions

The skull base triangles allow the surgeon to access neurological lesions through corridors while recognizing key adjacent structures. Knowledge of these anatomical apertures and their respective dimensions are critical in the recognition of pathology and the avoidance of injury to vascular and nervous structures.
